# Supplementary material for: Viscoelastic extracellular matrix enhances epigenetic remodeling and cellular plasticity
Source: Nat Commun. 2025 Apr 30;16:4054. doi: 10.1038/s41467-025-59190-7 (PMC12043949; doi:10.1038/s41467-025-59190-7)
Supplement: Supplementary file 2 — Description of Additional Supplementary Information [file 41467_2025_59190_MOESM2_ESM.pdf]

Description of Additional Supplementary Files

File Name: Supplementary Movie 1
Description: Calcium imaging of iN cells on a 2-kPa elastic substrate.

File Name: Supplementary Movie 2
Description: Calcium imaging of iN cells on a 2-kPa viscoelastic substrate.

File Name: Supplementary Movie 3
Description: Fibroblasts expressing GFP-tagged laminA on 2 kPa elastic gels #1.

File Name: Supplementary Movie 4
Description: Fibroblasts expressing GFP-tagged laminA on 2 kPa elastic gels #2.

File Name: Supplementary Movie 5
Description: Fibroblasts expressing GFP-tagged laminA on 2 kPa slow-relaxing gels #1.

File Name: Supplementary Movie 6
Description: Fibroblasts expressing GFP-tagged laminA on 2 kPa slow-relaxing gels #2.

File Name: Supplementary Movie 7
Description: Fibroblasts expressing GFP-tagged laminA on 2 kPa fast-relaxing gels #1.

File Name: Supplementary Movie 8
Description: Fibroblasts expressing GFP-tagged laminA on 2 kPa fast-relaxing gels #2.

File Name: Supplementary Movie 9
Description: FRAP of euchromatic regions in Hoechst-labeled cells cultured on various substrates.

File Name: Supplementary Movie 10
Description: FRAP of heterochromatic regions in Hoechst-labeled cells cultured on various substrates.

File Name: Supplementary Movie 11
Description: FRAP of fibroblasts expressing GFP-tagged HP-1alpha cultured on various substrates.
